# The emulsifying and foaming properties of Amuniacum gum (*Dorema ammoniacum*) in comparison with gum Arabic

**DOI:** 10.1002/fsn3.1658

**Published:** 2020-06-04

**Authors:** Behzad Ebrahimi, Aziz Homayouni Rad, Babak Ghanbarzadeh, Mohammadali Torbati, Pasquale M. Falcone

**Affiliations:** ^1^ Department of Food Science and Technology Faculty of Nutrition and Food Sciences Tabriz University of Medical Sciences Tabriz Iran; ^2^ Department of Food Science and Technology Faculty of Agriculture University of Tabriz Tabriz Iran; ^3^ Department of Food Engineering Faculty of Engineering Near East University Nicosia Turkey; ^4^ Department of Agricultural, Food and Environmental Sciences University Polytechnical of Marche Ancona Italy

**Keywords:** amuniacum gum, emulsion, foam, particle size, surface activity

## Abstract

In this study, the emulsifying and foaming properties of a novel exudate gum from *Dorema ammoniacum* (AMG) were assessed in comparison with the well‐known gum Arabic from Acacia tree (GAC). The sunflower oil‐based emulsion (10% v/v) containing various concentrations (5%–15% *w*/*v*) of AMG and GAC was prepared. At all concentrations, AMG showed higher surface and interface activity than GAC. Increasing in AMG and GAC concentrations caused to increase and decrease in Z average, respectively. Overall, the GAC‐stabilized emulsion showed lower Z average and PDI value than the AMG‐stabilized emulsion during storage time. The sample containing AMG showed higher emulsion capacity and lower emulsion stability in comparison with the one containing GAC at all concentrations. The storage stability decreased and increased with increasing in AMG and GAC concentrations, respectively. After two‐week storage, the emulsions containing 10 and 15% AMG showed higher phase separation than those containing GAC; however, this was opposite about sample containing 5% AMG. At thermal, centrifuge, and freezing conditions, the emulsion containing 5% AMG indicated significantly higher stability than GAC samples; however, at higher concentration, opposite effect could be observed. The foaming capacity of the samples containing AMG increased from 81% to 93% by increasing gum concentration from 5% to 15%. The solutions containing AMG showed higher foam capacity than control samples (without gum) and those containing GAC at all concentrations. Increasing in AMG and GAC concentrations slightly improved foam stability, and the highest value (92%) belonged to 15% AMG solution.

## Graphical abstract

In the present study, the emulsifying and foaming properties of a novel exudate gum from Dorema ammoniacum (AMG) were assessed in comparison with the well‐known gum Arabic from Acacia tree (GAC). The sunflower oil‐based emulsion (10% v/v) containing various concentrations (5%–15% *w*/*v*) of AMG and GAC was prepared. At all concentrations, AMG showed higher surface and interface activity than GAC.

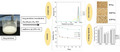

## 1 INTRODUCTION

Nowadays, hydrocolloids are used extensively in the food applications and they have different roles in food formulations such as thickening and gelling agents, stabilizer, emulsifying and foaming agents, coating and film formulations, fat replacer, antistaling, and crystallization inhibitor (Eduardo, Svanberg, & Ahrné, 2016; McClements, 2012). Mostly, functional properties of hydrocolloids are related to their water holding capacity and their ability in the improvement of rheological properties. Oil‐in‐water emulsions are widely used in food, cosmetic, and pharmaceutical formulations. They are thermodynamically unstable systems, because the contact between oil and water molecules is energetically unfavorable. Several types of instability such as creaming, Ostwald ripening, flocculation, and coalescence of the oil droplets can lead to phase separation in emulsion (Sun, Zhou, Sun, & Zhao, 2013). In the formulation of kinetically stable emulsion systems, two types of ingredient are usually used: emulsifying agents and stabilizers. Emulsifiers are surface active compounds that decrease surface tension and prevent droplet flocculation by adsorption on droplet surfaces. Stabilizers are hydrophilic biopolymers (proteins and polysaccharides), and they have ability of rheological modifications and induce long‐term emulsion stability.

Hydrocolloids can increase viscosity of continuous phase in colloidal solutions (sol, emulsions, and foams) that results in retarding the movement of particle, droplets, and bubbles, which in turn leads to enhancing of stability. On the other hand, a few of them act as surface active compounds and are used as emulsifying and foaming agents (Dickinson, 2009). Surface activity of polysaccharides normally depends to the presence of two types of chemical agents in their structure: (a) hydrophobic components such as methyl or acetyl groups attached to the hydrophilic polysaccharide backbone (in hydrophobically modified starch/cellulose, tragacanth, and pectin) and (b) existence of small fractions of proteins linked covalently or physically to the polysaccharide (gum Arabic, some type of pectin, etc.) (Dickinson, 2009). Gum exudates such as gum Arabic (GA), tragacanth, karaya, and Ghatti are amongst the oldest natural gums and have been used for thousands of years in the food and pharmaceutical products (Verbeken, Dierckx, & Dewettinck, 2003). Gum Arabic is the most convenient hydrocolloid, which is used as an emulsifier in emulsions, and it is commonly used at high levels (up to 20% of the total emulsion) in emulsions containing small quantities of oil (Nissim Garti & Leser, 2001). In practice, high gum/oil ratio (~1:1) is required to produce fine stable emulsion droplets (D4, 3 ≪ 1 µm), so it is expensive to be used (Dickinson, 2009). Emanuel et al. (2016) reported that exudate gum from *Prosopis alba is* able to stabilize emulsions better than GA based on its lower droplet size distributions, higher z‐potential, higher interfacial film elasticity, and lower values of the polydispersity and creaming indexes during storage. The study of Razavi, Cui, and Ding (2016) examined the surface activity of Balangu Shirazi and claimed that the surface tension of water at concentrations lower than 0.75% was reduced. In a recent study, Dabestani and Yeganehzad (2018) used a combination of Persian gum as an exudate gum with xanthan gum to have improving the foaming properties of the liquid egg white prior to pasteurization. The authors inferred that both hydrocolloids showed positive effects on foam stability; nevertheless, their negative effect on overrun and foam density was undeniable. Romdhane et al. (2017) studied the emulsifying and foaming properties of polysaccharide extracted from watermelon rinds (WMRP). Results showed that WMRP foam capacity and emulsifying property increased in a dose‐dependent manner at a concentration range of 1%–4% and 2%–4%, respectively.

Amuniacum gum is obtained from *Dorema ammoniacum D. Don (Umbelliferae)* exudate and also is locally known as Kandal, Vasha, and Koma‐kandal in Persia. This gum exists in yellow colors and is consumed traditionally for the treatment of some diseases such as anthelmintic, gastrointestinal disorders, antispasmodic, and expectorant (Amin, 2005). There has been no report about the structure–function relationship of this natural hydrocolloid. Therefore, the objective of current research study was evaluation of emulsifying and foaming properties of Amuniacum gum in comparison with gum Arabic.

## 2 MATERIAL AND METHODS

### 2.1 Materials

The AMG exudates were purchased from local herbal shops, Tabriz, Iran. For the comparison studies, commercial acacia gum (GAC) (generally referred as gum Arabic from Acacia tree) was purchased from Sigma‐Aldrich, USA (GA:G9752). Sunflower oil was purchased from local shop, Tabriz, Iran. Other reagents were provided by industrial manufacturers in analytical grades.

### 2.2 Extraction and purification of the AMG

Extraction and purification of the AMG was carried out based on a protocol by Saeidy et al. (2019) with modifications. Yellowish crude mucilage was milled to powder using blender. Extraction was carried out as dispersed mucilage in preheated distilled water with a water:mucilage ratio of 10:1 at a constant temperature of 50°C for 2 hr under mechanical stirring (200 rpm). The gum solution was stored overnight at 4°C for full hydration. Solution was centrifuged at 3,000 *g* for 30 min at 20°C, and then, the supernatant was collected, filtered (cloth filters), and precipitated using three volumes of 95% ethanol (*w*/*v*). Precipitation with ethanol removed lipophilic substances, pigments, and low molecular weight (LMW) proteins (Anvari et al., 2016). The sediment was collected, dried at room temperature, dissolved with 10 volumes of water (*w*/*v*), mixed with acetone for depigmentation, and precipitated with three volumes of ethanol (*w*/*v*) to increase its purity. The precipitate was collected as described previously and then was grounded and screened using mesh‐18 sieve. This was packed and stored at a cool dry condition**.**

### 2.3 Preparation of AMG and GAC solutions

In brief, AMG and GAC emulsions were prepared one day before emulsification by dissolving 5, 10, and 15% *w*/*v* of the gums in 60°C distilled water with a constant stirring for at least 12 hr using magnetic stirrer. Stock emulsions were stored at 4°C for at least 18 hr for complete hydration.

### 2.4 Preparation of the emulsions

Oil‐in‐water emulsions were prepared by adding 10% v/v drop‐wise of sunflower oil into the aqueous phase while stirring at 447.2 for 5 min using high‐speed stirrer (Fater, Iran). The emulsion was prepared by homogenization at  4472*g* for 6 min at alternate cycles of homogenization (1 min) and rest (2 min). The whole process was carried out using ice bath to minimize possible lipid oxidations. Then, 0.02% *v*/*v* of sodium azide was added to the samples as antimicrobial agent. Each sample emulsion was prepared at five various concentrations for surface and interfacial tension measurements. Concentrations included 15.00, 10.00, 5.00, 1.50, 1.00, and 0.5% *w*/*v* for AMG and GAC emulsions. Emulsions were prepared by dissolving samples in distilled water at 70°C for 12 hr with constant stirring followed by cooling at room temperature with stirring for 2 hr before the measurements.

### 2.5 Methods

#### 2.5.1 Analysis of the chemical compositions

Crude fat, ash, moisture, uronic acid, and carbohydrates of the AMG and GAC emulsions were analyzed using an original method by Karazhiyan et al. (2009). Protein contents (%) were analyzed using elemental analyzer (Elementar, Vario EL Series III, Germany) by multiplying nitrogen contents (%) by 6.25 (Kang et al., 2011). Mineral contents (Na^+^, K^+^, and Ca^2+^) were analyzed using PerkinElmer Optima 7300DV ICP‐OES (Shelton, CT, USA). The MWs of AMG and GAC emulsions were calculated using high‐performance size‐exclusion chromatography (HPSEC) (Shimadzu SCL‐10Avp, Shimadzu Scientific Instruments) (Wang, Wood, Huang, & Cui, 2003). Chemical composition analyses of the AMG and GAC emulsions were carried out thrice.

#### 2.5.2 Surface and interfacial tensions

Surface tension of the air–water interfaces was assessed based on a method described by Izydorczyk, Biliaderis, and Bushuk (1991). Decreases in surface tension (mN/m) of water with increases in concentrations of the samples were assessed using Wilhelmy plate method and tensiometer (Attension Sigma 702, Biolin Scientific). Forces on the ring were measured as it moved upward from an air‐gum dispersion surface. Equal volumes of the emulsions (50 ml) were transferred into 100‐ml glass beakers. Changes in the surface tension were recorded every 10 min at 22.0°C ± 0.5 for 2 hr. Values of the surface tension included the average of 12 measurements from 10 to 120 min. Sunflower oil was used for interfacial tension assessments. The oil was added to a constant volume of the aqueous gum emulsion and set for 3 min. The force needed to pull the Wilhelmy plate from one phase to another one was equal to interfacial tension. All assessments were carried out at 25°C.

#### 2.5.3 Droplet size, polydispersity index, and zeta‐potential

Droplet size distribution of the emulsions was studied at 25°C using dynamic light scattering instrument (Zetasizer Nano‐ZS90, Malvern Instruments), immediately after preparation and subsequently at 25°C on days 3, 7, 14, and 30. The studies were carried out after appropriate dilutions of the emulsion samples using Milli‐Q ultrapure water to avoid multiple scattering effects (Gu, Decker, & McClements, 2007). The Z‐average diameter of the emulsion droplets, z‐potential, and polydispersity index (PdI) were automatically calculated by the instrument as mean of ten reading values per sample. Refractive index values used for dispersed and continuous phases included 1.52 and 1.33, respectively.

#### 2.5.4 Optical microscopy

Microstructure of the emulsions was visualized using optical microscope (Leica Microsystems) equipped with a camera. Emulsions were gently agitated in glass test tubes before analysis to ensure their homogeneity and then were diluted using sodium dodecyl sulfate (1:40). A droplet of emulsion was transferred onto a microscope slide, covered by a cover‐slip, and studied using digital USB camera linked to a light microscope. Images were recorded at 100× objective magnifications**.**

#### 2.5.5 Capacity and stability of the emulsions

##### Emulsifying capacity

Fresh emulsions were centrifuged at 135.278*g* for 10 min. The emulsifying capacity (EC) was calculated based on the following formula by Jindal, Kumar, Rana, and Tiwary (2013a):

$$\text{EC}=\frac{f_{\mathrm{ev}}}{I_{t}}\times 100$$

where *f*_ev_ was the final emulsion volume and *I*_t_ was the total volume.

##### Stability during storage

A 10‐ml freshly prepared emulsion was transferred into a test tube, and the tube was capped and stored at 25°C. Stability of the emulsion was monitored immediately after the preparation and subsequently on days 3, 7, 14, and 30 of storage. During storage, samples were separated to opaque cream layers at top and transparent serum layers at bottom. Then, initial height (*H*_i_) and final height (*H*_f_) of the emulsions were measured. Stability was expressed as the ratio of height of the final emulsion to initial height of the emulsion in tubes (Koocheki, Kadkhodaee, Mortazavi, Shahidi, & Taherian, 2009; Nikiforidis & Kiosseoglou, 2007).

$$\text{ES}=\frac{H_{f}}{H_{i}}\times 100$$

##### Centrifuge stability measurement

Centrifugation method was used for ES by centrifugation 10 ml of emulsions at 1369*g* for 15 min. The ES was calculated as follows:

$$\text{ES}=\frac{f_{\text{ev}}}{I_{\text{ev}}}\times 100$$

where *f*_ev_ was the final emulsion volume and *I*_ev_ was the initial emulsion volume (Sciarini, Maldonado, Ribotta, Perez, & Leon, 2009).

##### Thermal stability

Emulsions were heated at 80°C for 30 min using water bath and then centrifuged at 3,500 rpm for 10 min. The ES was calculated as follows:

$$\text{EC}\;=\;\frac{f_{\text{ev}}}{I_{\text{ev}}}\;\times \;100$$

where *f*_ev_ was the final emulsion volume and *I*_ev_ was the initial emulsion volume (Bi, Yang, Fang, Nishinari, & Phillips, 2017).

##### Freeze–thaw treatment

Effects of Freeze–thaw cycles on emulsions were assessed using a method previously described by Gu et al. (2007). Emulsion samples (10 ml) were incubated at −20°C for 22 hr. Then, samples were incubated at 40°C for 2 hr. This Freeze–thaw cycle was repeated tree times with creaming stability comparisons between the emulsion samples.

#### 2.5.6 Foam characterization

The AMG and GAC dispersions were prepared at 5%, 10%, and 15% *w*/*v* concentrations and stored at 4°C overnight. After complete gum hydration, 1% of ovalbumin (Applichem) was added to the samples and agitated vigorously for 4 min. Immediately after agitation, foam was gently transferred to a measuring cylinder (100 ml) and weighed. The drained liquid was measured after 30 min. Foam, stability, and density values were calculated using the following equations:

$$\text{Foam}\;\text{Capacity}\;\left(\%\right)=\frac{i_{\text{fv}}}{t_{\text{sv}}}\times 100$$

where *i*_fv_ was the initial foam volume and t_sv_ was the total suspension volume;

$$\text{Foam}\;\text{Stability}\;\left(\%\right)=\frac{f_{\text{fv}}}{i_{\text{fv}}}\times 100$$

where *f*_fv_ was the foam volume after 30 min and *i*_fv_ was the initial foam volume; and

$$\text{Foam}\;\text{Density}=\frac{m_{100f}}{v_{100f}}\times 100$$

where m_100f_ was weight of 100 ml of foam (g) and V_100f_ was volume of 100 ml of foam (ml). All samples were prepared four times (Bovskova and Mikova, 2011; Dabestani & Yeganehzad, 2018; Jarpa‐parra, Tian, Temelli, Zeng, & Chen, 2016).

### 2.6 Statistical analysis

Data statistical analysis was carried out using ANOVA method. Furthermore, Duncan's post hoc comparison was used with a significance level of *p* < .05. Data were analyzed using SPSS Software v.17.0 (SPSS Inc.). Charts were drawn using Origin 2018 Software (Origin Lab).

## 3 RESULTS AND DISSECTION

### 3.1 Chemical composition

The protein and carbohydrate contents of gums directly affect their emulsifying properties. The chemical composition of AMG and GAC powders used in this study is shown in Table 1. The AMG had lower carbohydrates and lower protein contents in comparison with GAC. The amount of the total carbohydrate of other gum exudate, for example, *Persian gum* (87.17%), *Prunus armeniaca* (66.89%), *Prunus Cerasus* (71.51%), and anghouzeh gum (85.4%) was higher than AMG (Fathi, Mohebbi, & Koocheki, 2016b; Golkar, Taghavi, & Dehnavi, 2018; Milani, Ghanbarzadeh, & Maleki, 2012). Higher carbohydrate content represents higher purity of hydrocolloids (Golkar et al., 2018). AMG had 3.6% ash content that was close to it in GAC (3.4%). Moreover, the results indicated that fat contents of AMG were higher than GAC. The quantities of the different minerals of AMG in this study are as follows: calcium˃ potassium ˃ sodium. Factors such as different source of gums, age of trees, and the growth conditions, different purification methods, and period of exudation lead to this different chemical composition of hydrocolloids (Fathi, Mohebbi, & Koocheki, 2016a).

**Table 1:** Chemical composition of GAC and AMG

Composition (%)	Gum type	
	GAC	AMG
Moisture	11.4 ± 0.15^b^	12.10 ± 0.10^a^
Protein	1.9 ± 0.07^a^	0.3 ± 0.2^b^
Fat	1.15 ± 0.12^b^	17.8 ± 0.7^a^
Ash	3.4 ± 0.2^a^	3.6 ± 0.13^a^
Total carbohydrate	89.9 ± 0.41^a^	60.05 ± 0.05^b^
Molecular weight (KDa)	932	35
calcium (ca^2+^)	1.07 ± 0.01^b^	5,250.3 ± 0.5^a^
Potassium (K^+^)	0.2 ± 0.5^b^	875.03 ± 0.05^a^
Sodium (Na^+^)	0.4 ± 0.5^b^	91.1 ± 0.1^a^

Note

All measurements were carried out in triplicate, and the results are reported as the mean ± *SD*.

Similar letters (within the row) are not significantly different at *p* < .05.

### 3.2 Surface and interface activity

**Figure 1:**
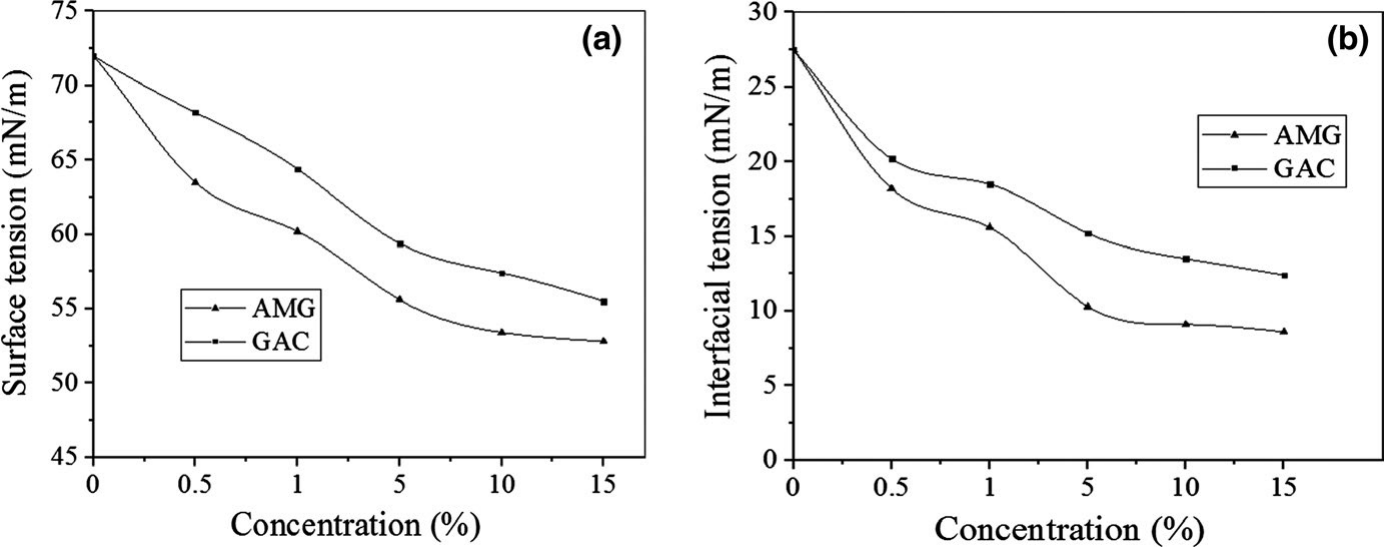
Surface tensions of aqueous solutions (a) and interfacial tensions of water–sunflower oil (b) in the presence of AMG and GAC at various concentrations (25°C)

Results of surface tension assessments of the AMG and GAC solutions are shown in Figure 1a. Surface tensions of both polysaccharide solutions were concentration‐dependent, and it decreased with increasing of polysaccharide concentration. A sharp decrease was seen in both solutions from 0.5% to 5%, which shifted to a plateau when the concentration was greater than 5%. The surface tension decreased from 72.5 mNm^−1^ to 52 and 57 mNm^−1^ by AMG and GAC at 15% concentration, respectively.

Timilsena, Adhikari, Kasapis, and Adhikari (2016) suggested that at higher concentrations, surface active biopolymers have lower freedoms to move due to steric phenomenon and they migrate with difficulty to air–water surfaces. Upon increasing quantities of the hydrocolloids, diffusional transport to the air–water surface decreases due to the chain overlap or molecular flocking, which results less decrease in surface tension per unit of the gum concentration. At all concentrations, AMG showed higher surface activity than GAC, and as a result, lower surface tension could be observed which it could be attributed to lower molecular weight of AMC than GAC or having higher hydrophobic groups such as methyl on its backbone (Dickinson, 2003). Koocheki, Taherian, and Bostan (2013) and Naji‐tabasi, Mohammad, and Razavi (2016) indicated that tendency to adsorb at air–water surface increased with decreasing of molecular weight of polysaccharides. Molecular weight has direct influence on viscosity. At the same concentration (5%), the viscosity of the emulsions was 0.1 and 0.3 Pa.s for AMG and GAC samples at a shear rate of 10 s^−1^. Garti, Slavin, and Aserin (1999) reported that most of gums decrease surface tensions, ranging from 65 to 42 mN/m. At 0.5% (*w*/*w*) concentrations of xanthan, carrageenan, methylcellulose, guar, pectin, and fenugreek, surface tension decreased to 60, 65, 52, 55, 53, and 50 mN/m, respectively (Huang, Kakuda, & Cui, 2001), which were lower than that at 0.5% AMG solution (63.5 mN/m). The 1.5% yellow mustard mucilage and pectin solution decreased surface tensions to 47 and 55 mN/m, respectively, which were lower than that in 1% AMG solution (60.2 mN/m) (Wu, Eskin, Cui, & Pokharel, 2015).

Results of interfacial tension measurements in the sunflower–water emulsion containing AMG and GAC are presented in Figure 1b. Interfacial activity of both gums was similar to behaviors observed for the surface tension. The oil–water interfacial tension decreased from 27 to 8.6 and 12.4 mN/m in the presence of 15% AMG and GAC, respectively. GAC was interfacially less active than AMG.

Polysaccharides are mostly hydrophilic polymers, which do not expect to exhibit significant surface activities (Dickinson, Murray, Stainsby, & Anderson, 1988). However, some polysaccharides can slowly migrate to air–water or oil–water interfaces and exhibit surface and interface activities (Garti et al., 1999). The surface activity of gum Arabic is related to its proteinaceous moieties (Chanamai & McClements, 2001). Osano, Hosseini‐Parvar, Matia‐Merino, and Golding (2014) demonstrated that decreasing of proteins levels in basil seed gum significantly decreased its surface active properties. The ability of gums in decreasing of surface and interfacial tension affects droplet size and emulsion stability; however, it is not only effective factor on these properties, and other factors such as effect of hydrocolloid on rigidity of surface layer, amount of depletion force, and viscosity are also important.

### 3.3 Droplet size distribution and polydispersity index (PDI)

**Figure 2:**
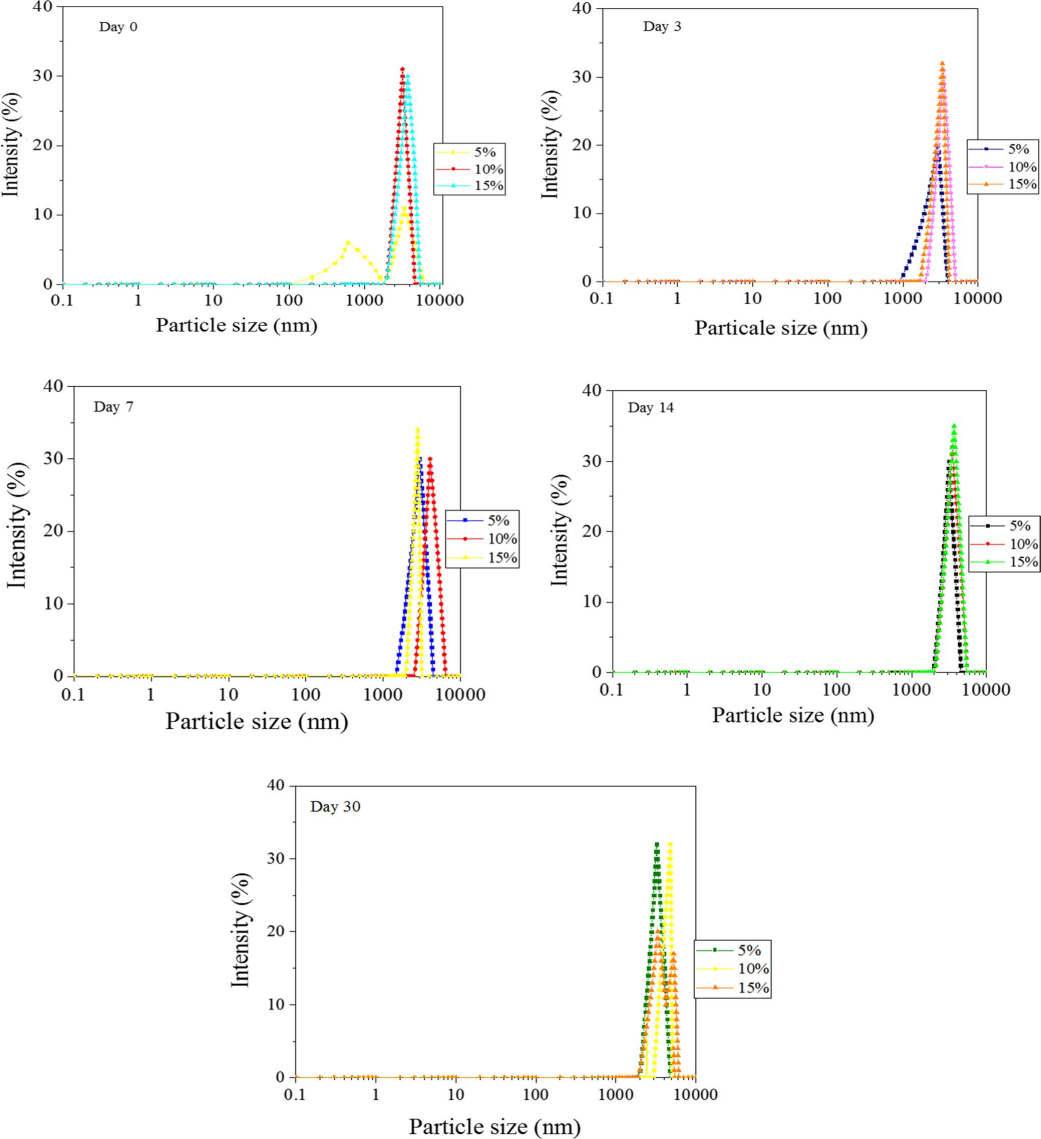
Intensity droplet size distributions of the emulsions containing AMG after 30 days

**Figure 3:**
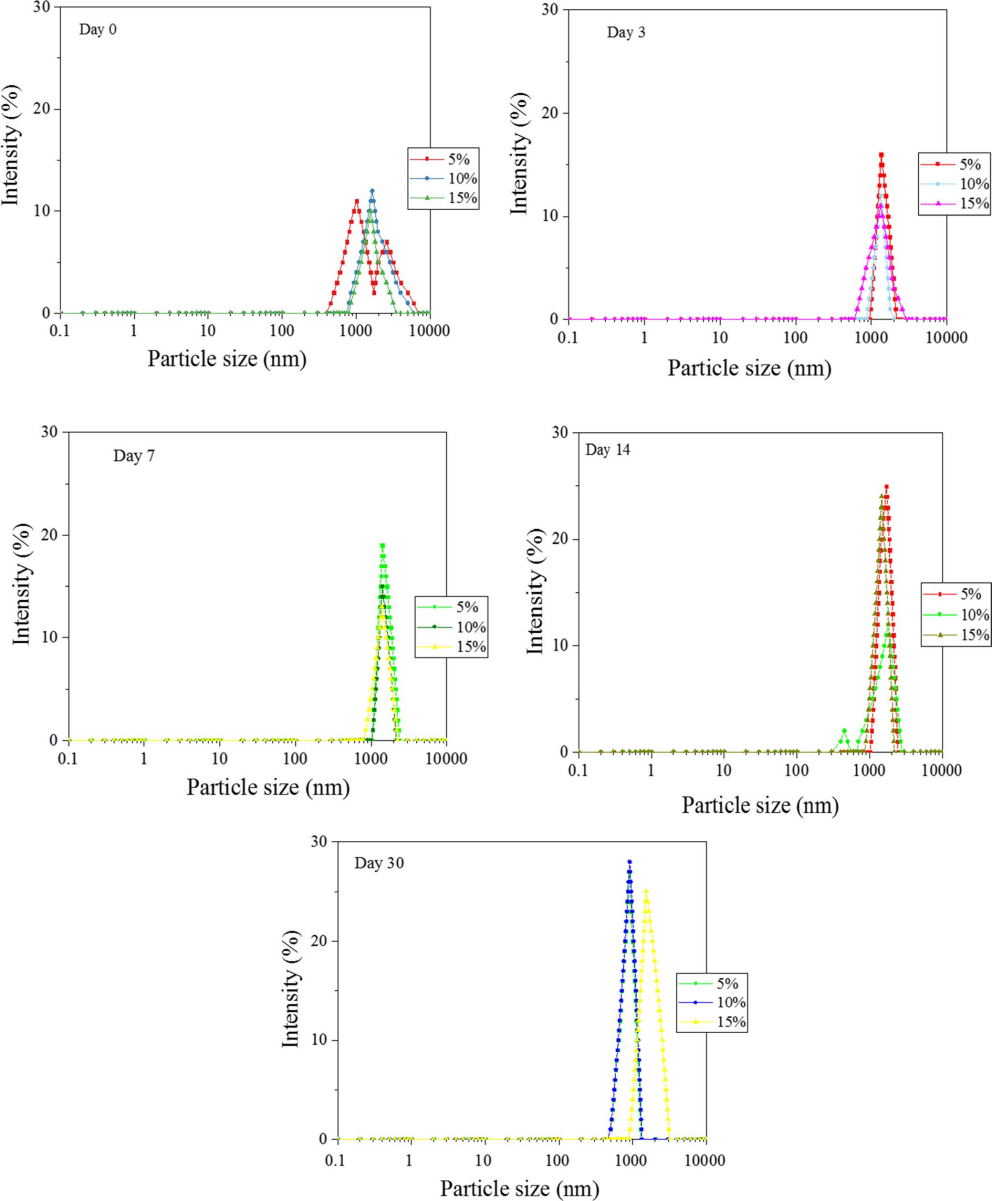
Intensity droplet size distributions of the emulsions containing GAC after 30 days

Emulsions with lower droplet sizes show higher stability because creaming rate is proportional to the square of the droplet diameter, and hence, a decrease in droplet size decreases the rate of gravitational separation (Golkar et al., 2018). Figures 2 and 3 show droplet size distributions in the emulsions containing AMG and GAC. The results showed that in the emulsions containing AMG gums, the peak slightly moved toward higher size values with increasing the gum concentration. Similarly, Vicente, Pereira, Bastos, de Carvalho, and Garcia‐Rojas (2018) reported that the average particle size distribution increased with increasing pectin or xanthan gum concentration. Bimodal distribution was observed in the AMG stabilized emulsions immediately after preparation and on day 30 at 5% and 15% AMG concentrations, respectively (Figure 2). This behavior was observed for the GAC stabilized emulsions immediately after preparation and on day 14, at 5% and 10% GAC (Figure 3). Similarly, bimodal distribution was observed by Huang et al. (2001) for the emulsion containing xanthan and by Osano et al.(2014) for a new polysaccharide from basil seeds (*Ocimum bacilicum* L.) at 0.3% *w*/*v* gum concentrations. McClements (2007) reported that bimodal droplet distribution systems were less stable than monomodal ones due to Ostwald ripening mechanism. The PDI value is extremely important stability parameter because it provides indications on wideness of the droplet size distribution. In the current study, all emulsions were polydispersed emulsions (PDI ˃ 0.2) (Table 2). The PDI value of the emulsion containing 5% AMG was relatively low over time (0.11–0.43); however, the AMG‐stabilized emulsions showed higher PDI values at higher concentrations, which indicates higher tendency for destabilization. Increase in polydispersity indices with increasing of concentrations of xanthan or pectin in emulsions has been also reported by Vicente et al. (2018). In contrast to AMG‐stabilized emulsion, the PDI values decreased with increasing concentration in GAC‐stabilized emulsions (from 0.6 to 0.26 at first day). The low PDI value shows faster kinetics of emulsifier adsorption immediately after emulsion preparation, which inhibits from re‐coalescence and widening of the distribution (Mahfoudhi et al., 2014). The PDI values of the AMG‐ and GAC‐stabilized emulsions decreased and increased over time, respectively.

**Table 2:** The average particle size (µm) and polydispersity index (PDI) of emulsions prepared with AMG and GAC as a function of gum concentration and storage time

Days	Gum concentration (%)	Z‐average (µm)		PDI	
		AMG	GAC	AMG	GAC
0	5	1.32 ± 0.5^c^	1.35 ± 0.01^a^	0.33 ± 0.05^b^	0.6 ± 0.01^a^
	10	3.06 ± 0.01^b^	1.32 ± 00^b^	0.65 ± 0.05^a^	0.30 ± 0.01^b^
	15	3.5 ± 0.04^a^	1.30 ± 0.02^c^	0.55 ± 0.04^a^	0.26 ± 0.01^c^
3	5	1.8 ± 0.05^b^	1.38 ± 0.01^a^	0.43 ± 0.01^c^	0.37 ± 0.02^a^
	10	3.14 ± 0.01^a^	1.35 ± 0.05^b^	0.60 ± 0.01^a^	0.33 ± 0.02^b^
	15	3.1 ± 0.1^a^	1.32 ± 0.03^c^	0.52 ± 0.01^b^	0.33 ± 0.03^b^
7	5	2.33 ± 0.1^c^	1.40 ± 0.02^c^	0.31 ± 0.01^b^	0.43 ± 0.01^a^
	10	3.5 ± 0.05^a^	1.36 ± 0.1^a^	0.53 ± 0.01^a^	0.39 ± 0.03^b^
	15	2.6 ± 0.05^b^	1.34 ± 0.01^b^	0.21 ± 0.01^c^	0.38 ± 0.01^b^
14	5	2.4 ± 0.05^c^	1.98 ± 0.06^c^	0.11 ± 0.02^c^	0.56 ± 0.02^a^
	10	3.7 ± 0.1^a^	1.89 ± 0.04^a^	0.51 ± 0.04^a^	0.55 ± 0.01^a^
	15	2.9 ± 0.05^b^	1.85 ± 0.02^b^	0.26 ± 0.03^b^	0.50 ± 0.03^b^
30	5	2.8 ± 0.01^c^	2.72 ± 0.1^c^	0.01 ± 0.01^c^	0.58 ± 0.01^a^
	10	4.4 ± 0.17^a^	2.55 ± 0.02^b^	0.49 ± 0.03^a^	0.59 ± 0.01^b^
	15	3.2 ± 0.05^b^	2.05 ± 0.04^a^	0.31 ± 0.04^b^	0.38 ± 0.01^c^

Note

Values were means of triplicate values ± standard deviations.

Different letters (a–c) in the same column indicate statistically significant differences at *p* < .05

The Z‐average is a useful mean diameter value when PDI value is low (usually below 0.7) and it is sensitive to the presence of large droplets. Table 2 shows the Z‐average of the oil droplets in the emulsions containing various AMG and GAC concentrations over storage time at ambient conditions.

For freshly prepared emulsions (day 0), the samples containing 5% AMG showed the lowest Z‐average (1.35 µm) and the mean particle size increased significantly with increase in AMG concentration. At higher AMG concentrations, depletion flocculation phenomena probably resulted formation of larger oil droplets. Similar trend reported by Jafari, Beheshti, and Assadpour (2013) who observed that increasing of Persian gum concentration at a range of 1%–5% increased Z‐average from 4.242 to 8.191 μm.

In contrast to AMG, increasing in GAC concentration decreased Z‐average, which can be attributed to further gum adsorption to interface that lead to more decreasing of interface tension and more repulsive forces on the surface of droplets. These results are similar to those reported by previous studies (Liu et al., 2018; Tzoumaki, Moschakis, Kiosseoglou, & Biliaderis, 2011; Xu et al., 2016; Zhuang et al., 2016). With increasing the GAC concentration, a higher protein content is available to cover oil droplets, which can inhibit droplet flocculation and coalescence and result in smaller particle sizes (Liu et al., 2018).

After one month, Z‐average increased in GAC emulsions at all concentrations. This was seen in AMG emulsions at 5% and 10% gum concentrations. Nakauma et al. (2008) found that the Z‐average increased in the emulsions containing sugar beet pectin (1.5%), soybean soluble polysaccharide (4.0%), and gum Arabic (10.0%) after storage for 3 days. Unlike this research, Hosseini, Jafari, Mirzaei, Asghari, and Akhavan (2015) reported that the Z‐average decreased for the stable emulsions containing gum Arabic after 48 hr of storage. In other research work, the Z‐average and PDI values decreased in the emulsions containing ovalbumin and pectin over 14 days of storage (Vicente et al., 2018). The Z‐average of the emulsions stabilized with β‐lactoglobulin–acacia seyal gum conjugates decreased from 10 to 6.7 µm with increasing incubation time (Bi et al., 2017).

The surface load (*Γ*_s_) is a further reliable mean of estimating the minimum emulsifier quantity necessary to cover all droplets (Mcclements, 2007):

$$\Gamma =\frac{d_{3,2}\times C_{s}}{6\times \varphi }$$

where C_s_ is the concentration of emulsifier in the emulsion (kg/m^3^), Γ is the surface load of the emulsifier at saturation (kg/m^2^), φ is the dispersed phase volume fraction, and d_3,2_ is the mean volume‐surface droplet diameter produced by the homogenizer. This equation assumes that all emulsifier molecules are extremely quickly adsorbed to the droplet surfaces during homogenization, which is unlikely to occur for large molecules like polysaccharides in experiments. Therefore, calculated surface loads can be considered as effective values and used for comparing relative effectiveness of various emulsifiers in commercial uses (Bai, Huan, Li, & Julian, 2017). In this study, Equation 1 was used to calculate surface loads of the polysaccharides based on the oil droplet concentration (φ = 0.1), emulsifier concentration (5%–15% *w*/*v* or 50–150 kg/m^3^), and measured mean droplet diameters (d_3,2_). Calculated surface loads of the AMG and GAC emulsions included 86.6 and 88.6 mg/m^2^, respectively. This might be attributed to differences in MW and packing of the emulsifier molecules at oil–water interfaces. Values reported for gum Arabic, beet pectin, and corn fiber gum were, respectively, reported by Bai et al. (2017) as 31, 4.5, and 352 mg/m^2^. The current results were greater than the results of gum Arabic and beet pectin and lower than the results of corn fiber gum. In fact, higher values indicated that a large fraction of the gum molecules still existed in continuous aqueous phase and was not adsorbed to the oil droplet surfaces. Another possible explanation for the differences in the emulsification properties of different polysaccharide emulsifiers can be their effects on rheology of the aqueous phase. The flow profile of homogenizer was affected by rheological properties of the aqueous phase and higher viscosity to make the droplet disruption processes harder; hence, droplet sizes became larger.

### 3.4 The zeta‐potential

The z‐potential values of the emulsions immediately after preparation are reported in Table 3. Results showed similar behaviors for AMG‐ and GAC‐stabilized emulsions in terms of variations in z‐potential as a function of concentration which varied from −17.2 to −11.1 and −19 to −18.3 mV, respectively. However, the absolute z‐potential of GAC emulsions was significantly higher than that of AMG emulsions, which could be consider as a factor contributing to stability of the GAC emulsions. In the present study, negative surface charge of the emulsion droplets decreased with increased gum concentration. Similar results were reported by Emanuel et al. (2016), who indicated that increased concentration of exudate gum Acacia from 2% to 5% decreased negative surface charge of the emulsion droplets (−41.20 to −36.10 mV for 2%–5% w/v Acacia‐stabilized emulsions). A possible explanation for this drop in z‐potential might be that by the addition of AMG and GAC surface pressure has caused denser packing of chains extending from the droplet surface thereby resulting in a less negative charge (Chivero, Gohtani, Yoshii, & Nakamura, 2015). Higher flocculation phenomena have been reported in emulsions prepared with acidic polysaccharides with low z‐potentials (z‐potential < |−30| mV) (Guo et al., 2014). In this study, z‐potentials of all emulsions were lower than |−30| mV, indicating low role of electrostatic repulsion in stability of these emulsion systems. Wu, Eskin, and Cui (2015) showed that when z‐potential is small, the interfacial activity and emulsifier concentration for full surface coverage are important.

**Table 3:** Zeta‐potential of emulsions prepared with AMG and GAC at different concentrations

AMG				GAC		
5%		10%	15%	5%	10%	15%
z‐potential (mV)	−17.2^a^	−14.2^b^	−11.1^c^	−19^a^	−17.4^c^	−18.3^b^

Note

Different letters (a–c) in the same raw indicate statistically significant differences at *p* < .05.

### 3.5 Optical microscopy

**Figure 4:**
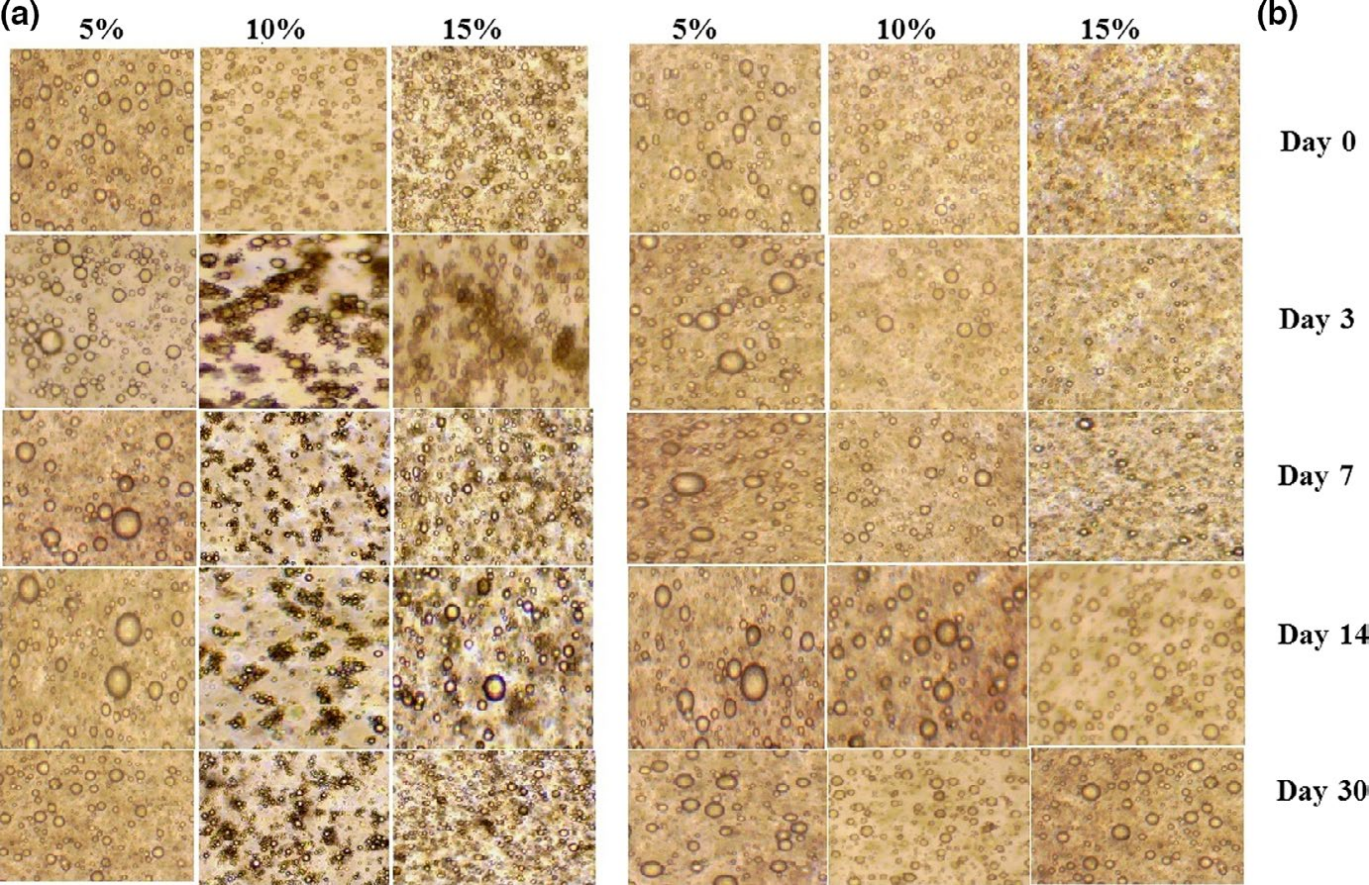
The morphology of the emulsions containing AMG (a) and GAC (b). Images of the emulsion microstructure were acquired using a 100 × objective lens

Figure 4 shows effects of AMG and GAC concentrations on morphology of droplets in the emulsions. Significant structural differences were observed between the emulsions as functions of the AMG and GAC concentrations. In the emulsions containing GAC, instability and extensive flocculation was not observable at all concentration of gum and increasing of concentrations induced further droplet size uniformity. These are similar to the results of Z‐average values obtained from DLS method (Table 2). The emulsion containing 5% AMG was relatively stable; however, at 10% and 15% AMG concentrations, extensive flocculation has been occurred during three days storage, which could be attributed to depletion flocculation mechanism.

### 3.6 Emulsion capacity and stability

Comparisons between the emulsion capacities of the formulations containing AMG and GAC are shown in Table 4. The sample containing AMG showed better emulsion capacity in comparison with the one containing GAC at all three used concentrations (5%, 10%, and 15%). Jindal, Kumar, Rana, and Tiwary (2013b) demonstrated higher emulsion capacity of bael fruit gum at 0.75% *w*/*v* concentration in comparison with gum Arabic.

**Table 4:** Emulsion capacity of AMG and GAC

Concentration (% *w*/*v*)	AMG	GAC
5	65.24 ± 2.5	60.43 ± 2.12
10	72.26 ± 3.21	63.22 ± 3.17
15	89.85 ± 1.54	72.20 ± 1.60

**Figure 5:**
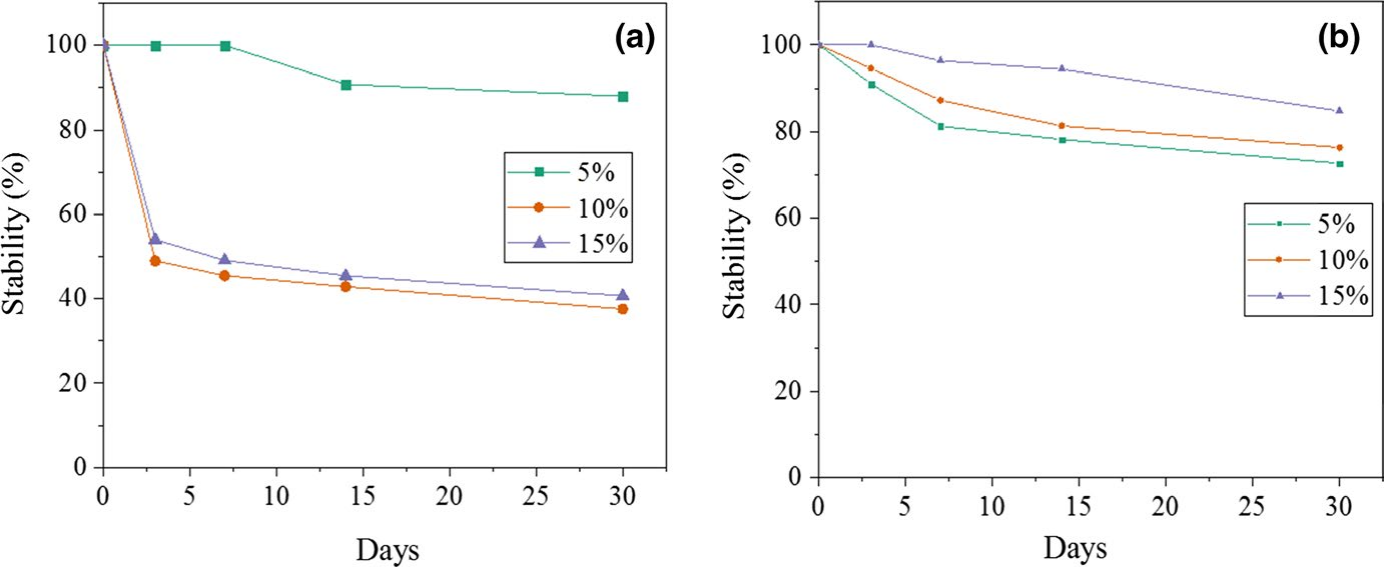
Stability (%) of emulsions containing AMG (a) and GAC (b) in different concentrations, during storage at 25 ˚C

**Figure 6:**
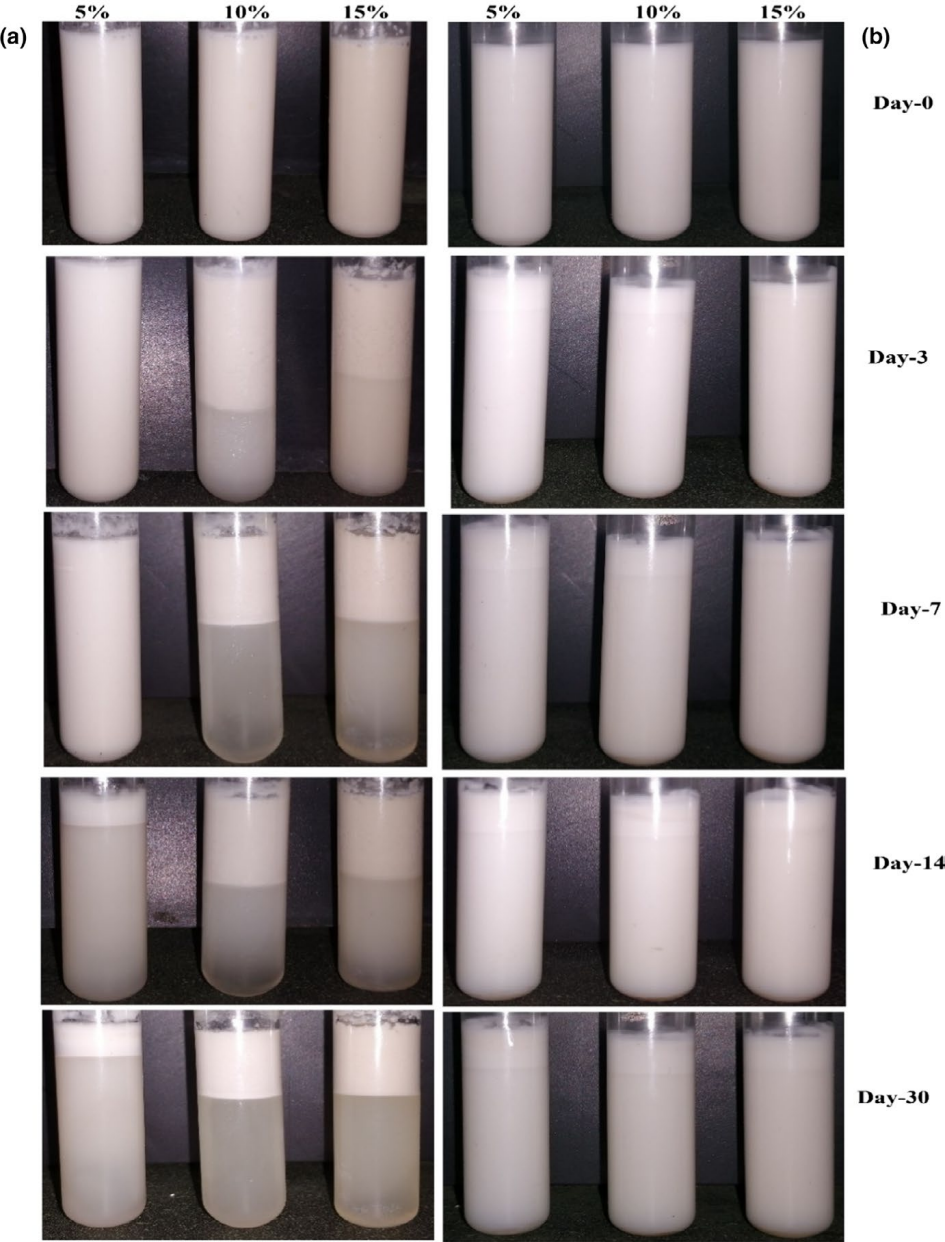
Visual appearance of emulsions stabilized by different concentrations of (a) AMG and (b) GAC. All the emulsions were stored at room temperature (25 ˚C)

Figure 5 shows effects of AMG and GAC at three concentrations on the stability of emulsions during 30‐day storage at 25°C. As can be seen, the stability decreased and creaming increased with increasing of AMG concentration from 5% to 15%. Unlike the emulsions containing AMG, the stability increased with increasing of concentration in those containing GAC. This could be attributed to growth of droplet size due to depletion flocculation by nonabsorbed hydrocolloid in the emulsion containing AMG at relatively high polysaccharide concentrations or decreasing of homogenization efficiency due to increasing of viscosity (McClements, 2012). A similar behavior has been reported for corn fiber gum, in which visible creaming was observed at high polysaccharide levels (Bai et al., 2017). However, opposite behavior has been observed in some other hydrocolloids AMG gum has very low molecular weight (35 KDa) and cannot greatly increase viscosity in continues phase and so depletion flocculation mechanism can predominate on stabilizing mechanism at 10% and 15% concentration. Figure 6 shows appearance of the emulsions stabilized by different concentrations of AMG and GAC stored at room temperature (25°C). In the emulsions containing 5% AMG, phase separation occurred after 14 days of storage; however, this time was three days for those containing 10% and 15% of AMG. In all days of storage, the emulsions containing 15% AMG were significantly more stable than those containing 10% AMG (*p* ˂ .05). At higher hydrocolloid concentrations, creaming is retarded due to the high viscosity and forming of gel network in continues phase of emulsion (Dickinson, 2009; Li, Sun, & Yang, 2012). Similar behavior in emulsion containing xanthan gum has been previously reported by Vicente et al. (2018). Creaming occurred on 3th day in the emulsions containing 10 and 15% AMG; however, it occurred on 7th day in those containing 10 and 15% of GAC. In 14th, the emulsions containing 10% and 15% AMG showed higher phase separation than those containing GAC; however, this was opposite about sample containing 5% AMG. This can be attributed to higher molecular weight or presence of higher protein moiety in GAC in comparison with AMG. The effect of polysaccharides on emulsion stability depends on two important parameters: (a) their ability in modification of continues phase rheology (b) and their ability in adsorbing to interface and covering of droplets. The viscosity of continues phase plays an important role in stability of colloidal systems such as foams and emulsions, and hydrocolloids can increase viscosity and forming gel network, which in turn lead to slowing down of droplets movement and decreasing of water drainage during droplet collision (Dickinson, 2009; Martinez, Baeza, Millan, & Pilosof, 2005; Ruiz‐Henestrosa, Martinez, Sánchez, Patino, & Pilosof, 2014). On the other hand, previous researchers have suggested that the presence of hydrophilic carbohydrates and hydrophobic proteins simultaneously in hydrocolloid structures (protein–polysaccharide complexes) such as gum Arabic confers an amphiphilic character to the gum allowing its adsorption at the oil–water interface and covering of droplet surface. This can decrease interfacial tension and hence increase homogenization efficiency in production of smaller droplets. On the other hand, thick protective biopolymer layer in newly formed interface can prevent from re‐coalescence after homogenization and during storage. The smaller droplets can potentially induce higher stability in emulsion and lower creaming phenomena (Qian, Cui, Wang, Wang, & Zhou, 2011). GAC has higher molecular weight than AMG; hence, it would have more effect on viscosity. On the other hand, it can cover droplet surface due to higher protein moiety content, which in turn prevent better from re‐coalescence (Brummer, Cui, & Wang, 2003; Dickinson, 2003).

**Figure 7:**
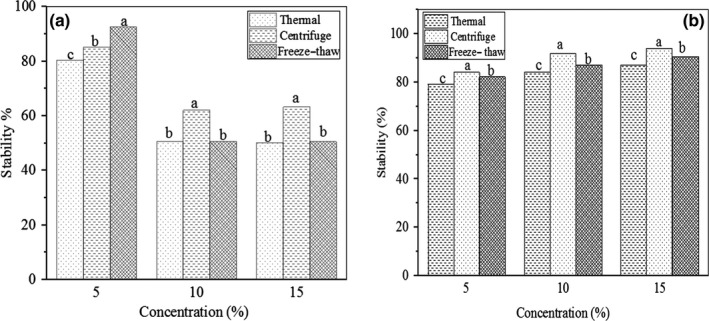
Thermal, centrifuge, and freeze–thaw stability tests of the emulsions containing AMG (a) and GAC (b) at different concentrations. For each parameter (thermal, centrifuge, and freeze–thaw), similar letters for each concentration are not significantly different at *p* < .05

Figure 7 shows stability of the emulsions at different concentrations of AMG and GAC against thermal, centrifuge, and freezing conditions. At these three conditions, the emulsion containing 5% AMG indicated significantly higher stability than those containing 10% and 15% AMG. On the other hand, this 5% AMG‐stabilized emulsion was more stable in freeze–thaw stability test than thermal and centrifugal experiments. However, the 10% and 15% AMG emulsion samples showed higher centrifugal stability. Heating, centrifuge, and freezing–thawing process can increase frequency of collision and destroy protective films around the droplets, which in turn lead to flocculation and coalescence (Sathivel, Huang, & Prinyawiwatkul, 2008). No significant differences were seen for heat and freeze–thaw stabilities in 10% and 15% AMG emulsions (*p *˃ .05). At these three tests, the emulsions containing 5% AMG showed higher stability than those containing GAC; however, at higher concentration, opposite effect could be observed. The GAC‐stabilized samples had good stability at all concentrations and showed the highest stability against centrifugal condition. This shows 5% GAC was not sufficient for coverage of droplet surface or effectively increasing of viscosity.

In fact, thermal, centrifugation, and freeze–thaw assays showed that AMG‐stabilized emulsions at 5% concentration had acceptable higher stabilities compared to the GAC‐stabilized emulsions. According to interfacial activity data, AMG gum shows higher surface activity than GAC, which could be lead to higher emulsion stability due to better droplet coverage. Furthermore, the ability of hydrocolloids in increasing of viscosity had not great role in emulsion stability (Golkar et al., 2018). These results are similar to results by Golkar et al. (2018) for the emulsifying properties of Persian gum and Wu, Cui, Eskin, and Goff (2009) for the emulsifying properties of four commercial galactomannans of Fenugreek, Guar, Tara, and Locust Bean gums.

### 3.7 Foaming properties

**Figure 8:**
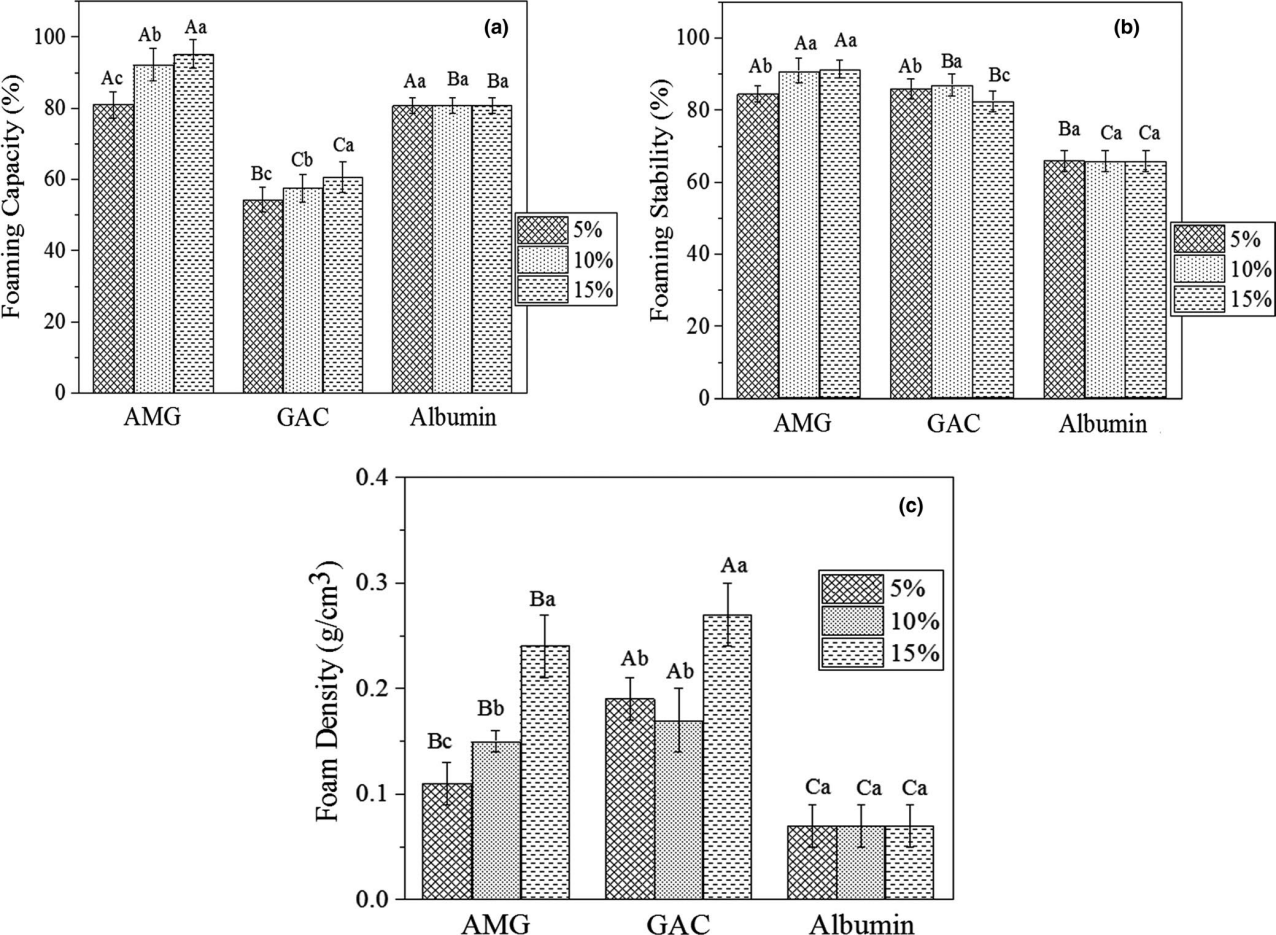
Effect of AMG and GAC on foaming capacity, stability, and density of 1% albumin solutions. Different letters of each bar indicate significant differences between gums (capital letter) and different gum concentrations (small letter) at *p* < .05

Foams can be described as dispersion systems with gaseous phases trapped in continuous matrices to form aerated structures in food materials (Marinova et al., 2009). Proteins and polysaccharides (as food macromolecules) play important roles in stabilization of foams by delaying drainage and creating a viscoelastic layer on the bubble surface, which protects films against mechanical disruption and prevents or retards Ostwald ripening (Jeddou et al., 2016). Figure 8 illustrates effects of various concentrations of AMG and GAC on foaming capacity of 1% albumin solutions. The foaming capacity of the samples containing AMG increased from 81% to 93% by increasing gum concentration from 5% to 15%. The solutions containing AMG showed higher foam capacity than control samples (without gum) and those containing GAC at all concentrations which may be related to lower molecular weight or higher hydrophobic groups in its structure which cause easier adsorption at the air–liquid interface. GAC decreased foam capacity of ovalbumin solutions; however, similar to AMG, increasing of GAC concentration increased the foam capacity.

Foam stability usually refers to ability of foams to sustain foam volumes and/or bubble sizes in a certain time period (Naji, Razavi, & Karazhiyan, 2012). Foam stability of the control albumin solutions was 66% and was improved by both AMG and GAC at all concentrations. Increasing in AMG concentrations slightly improved foam stability and the highest value (92%) belonged to 15% AMG solution. Various instabilities can be occurred in foam systems including gravitational separation, drainage, air‐bubble coalescence, mechanical disruption, and Ostwald ripening. Foam systems are subjected to phase separation due to density differences between the gas bubbles and continues phase (Jahanbin, Moini, Reza, Emam‐djomeh, & Masi, 2012). Air bubbles in foam systems have a tendency to approach and join together (coalescence). The result will be larger bubbles that are thermodynamically unstable and cause the foam system to collapse. Hydrocolloids prevent from these instabilities by modifying viscosity of aqueous phases, forming biopolymer gel networks, and forming viscoelastic layer on the bubble surface. The higher foaming stability in the AMG can be related to the presence of high content of hydrophobic groups, low MW, and flexible structure. Also, AMG and GAC showed surface activities that potentially could affect properties of the gas–liquid surface, including rigidity and permeability of the surface layer (Makri & Doxastakis, 2007).

The foams produced from pure albumin solutions showed low density (*ρ* = 0.07 g/m^3^) (Figure 8c). Addition of 5% AMG increased density of foam nearly twofold (*ρ* = 0.12 g/m^3^). Previous studies have demonstrated increase in foam density with adding hydrocolloids such as carrageenan and xanthan (Dabestani & Yeganehzad, 2018; Ptaszek et al., 2014). Hydrocolloids are thickener agents and create heavier foams than the foams produced from pure albumin (Ptaszek et al., 2014). In the present study, a significant increase was observed in density of foams containing GAC (*ρ* = 0.27 G m^−3^ for 15% GAC system). Binding of a large volume of water induces positive and negative effects on foaming properties; foam easily undergoes pumping and is resistant to destruction; however, if these products are dried, the high water content in their structures can be unfavorable (Ptaszek et al., 2014).

## 4 CONCLUSION

In this study, the emulsifying and foaming properties of ammoniacum gum were investigated in comparison with Arabic gum. AMG showed higher surface and interface activities also emulsifying capacity than Arabic one that could be attributed to low molecular weight and or having hydrophobic group in AMG backbone. However, generally the emulsion containing Arabic gum had lower particle size and higher stability during different conditions, which could be related to its higher protein content or higher molecular weight. The emulsion containing AMG gum showed smaller mean particle size and higher stability at the lowest used concentration (5% *w*/*w*). The AMG and GAC increased and decreased foaming capacity of albumin solution; however, both increased foam stability at all concentrations. As a result, it appears the AMG can be used as good foaming agent; however, it is not suitable by alone for long‐term stability of emulsions despite of its good emulsifying capacity.

## CONFLICT OF INTEREST

The authors declare that they do not have any conflict of interest.

## ETHICAL APPROVAL

Ethical Review: This study does not involve any human or animal testing.

## INFORMED CONSENT

Written informed consent was obtained from all study participants.
